# Clinical stage and histological type of the most common carcinomas diagnosed in young adults in a reference cancer hospital

**DOI:** 10.6061/clinics/2018/e656s

**Published:** 2018-09-10

**Authors:** Marina Candido Visontai Cormedi, Edia Filomena Di Tullio Lopes, Simone Maistro, Rosimeire Aparecida Roela, Maria Aparecida Azevedo Koike Folgueira

**Affiliations:** I Departamento de Radiologia e Oncologia, Instituto do Cancer do Estado de Sao Paulo (ICESP), Faculdade de Medicina FMUSP, Universidade de Sao Paulo, Sao Paulo, SP, BR; II Registro Hospitalar de Câncer, Instituto do Cancer do Estado de Sao Paulo (ICESP), Faculdade de Medicina FMUSP, Universidade de Sao Paulo, Sao Paulo, SP, BR

**Keywords:** Neoplasms, Young Adult, Neoplasm Staging, Histology

## Abstract

**OBJECTIVES:**

Cancer in young adults represents a great challenge, both biologically and socially, and understanding the unique characteristics of neoplasms in this age group is important to improving care. We aimed to evaluate the most common carcinomas and their characteristics, such as histological type and clinical stage, in young adults in the largest cancer hospital in Latin America.

**METHODS:**

The hospital registry was consulted for the period between 2008 and 2014. Young adults were defined as individuals aged 18 to 39 years, and older adults were defined as individuals aged 40 years and older. Differences between age groups were assessed through chi-square tests.

**RESULTS:**

Of the 39,389 patients included, 3,821 (9.7%) were young adults. Among the young adults, the most frequent cancer types were the following: breast, lymph node, colorectal, thyroid, testicle, hematopoietic and reticuloendothelial, uterine cervix, brain, soft tissue and stomach; these sites accounted for 74.5% of the observed tumors. Breast, colorectal and stomach cancers were more frequently diagnosed at advanced stages in young adults than in older adults (*p*<0.001). The most common histological types were infiltrating ductal carcinoma (86.12%) for breast cancer, adenocarcinomas not otherwise specified (45.35%) for colorectal cancer, squamous cell carcinoma not otherwise specified (65.26%) for uterine cervix cancer, signet ring cell adenocarcinomas (49.32%) for stomach cancer and adenocarcinomas not otherwise specified (50.79%) for lung cancer.

**CONCLUSION:**

Young adults are diagnosed with cancer at more advanced stages, indicating that health professionals should be aware of cancer incidence in this age group. It is necessary to develop a better understanding of cancer in young adults and to implement dedicated health care strategies for these patients.

## INTRODUCTION

Cancer is one of the main health problems among young adults (aged 20 to 39 years), and it is the second and fifth leading causes of death in women and men, respectively, in the USA 1. In addition, approximately 70,000 adolescents and young adults (AYA), aged 15 to 39 years, are diagnosed with cancer each year in the USA alone 2,3.

In young adults (YA), cancer is associated with unique issues including increased social and psychological morbidities, fertility preservation, schooling and work concerns, as well as long term side effects of treatment 4,5. In addition, young adults may be diagnosed with cancer at more advanced stages as a consequence of delayed cancer diagnosis, or unique biological characteristics 6. In the face of these many challenges, some agencies have proposed recommendations to help young adults with cancer regain a sense of control over their lives 7.

In general, the most common cancers in AYA, aged 15 to 39 years, are Hodgkin's lymphoma, melanoma, testicular carcinoma, germ cell carcinoma, cervical carcinoma, thyroid carcinoma, soft tissue sarcoma, and osteosarcoma. Some of these cancers are uncommon in other age groups, such as germ cell ovarian and testicular carcinomas. This distribution contrasts with that in young children, in whom small round cell embryonic tumors, such as neuroblastoma, Wilms' tumor, retinoblastoma, rhabdomyosarcoma and teratomas, are more common. AYA neoplasms also differ from those in middle-aged and elderly people, in whom malignancies of epithelial cell origin or carcinomas of several primary sites account for more than 85% of cancers 3. Nevertheless, some AYA develop certain carcinomas that are commonly diagnosed in older individuals but that are uncommon in AYA.

In Brazil, data from the Brazilian National Cancer Institute (Instituto Nacional do Câncer, INCA) 8 indicate that the most frequent neoplasms in AYA, aged 15 to 29 years, were carcinomas of all types (34.2%), followed by lymphomas (12.1%) and skin tumors. This study also reported a trend towards an increase in the average annual percent change (AAPC) during the reference period in the incidence rates of all neoplasms in this age group in four out of the 13 population-based cancer registries (Registros de Câncer de Base Populacional - RCBPs), mainly due to an increase in the carcinoma incidence rate, which has been observed in eight out of the 12 RCBPs. Regarding mortality rates, even though there was no significant variation in the AAPC considering all neoplasms in the whole reference period, there was a small decrease in the mortality rate from 1982 to 1983, followed by more recent increases in the mortality rate from 1992 to 1998 and from 1998 to 2013. In that study, however, data regarding the most frequent types of carcinomas in AYA and the clinical stages were not available.

Hence, unfortunately, in Brazil, there is a scarcity of data regarding some types of cancer in young adults, such as carcinomas. Our aim was to evaluate the most common primary sites of cancer as well as the histological type and clinical stage at diagnosis of frequent carcinomas in young adults who were treated at a main reference cancer hospital in Latin America, the Instituto do Câncer do Estado de São Paulo (ICESP).

## METHODS

This observational study was conducted at the ICESP in São Paulo City, Brazil. ICESP, the largest cancer hospital in Latin America, is integrated with the Brazilian public health system (Sistema Único de Saúde, SUS) and delivers care to patients referred from primary, secondary and non-oncologic tertiary centers.

Patient data were obtained on February 1^st^, 2018, from the hospital registry database. The period searched was from May 1^st^, 2008, to April 30^th^, 2014. All patients registered at the ICESP during that period with malignant neoplasms (International Code of Diseases, ICD C00 – C96) were included and considered for the evaluation of the primary site of disease, clinical stage and histological type. Patient gender was obtained for a subgroup of patients registered from January 1^st^, 2009, to December 31^st^, 2013. Patients with basal cell carcinoma and squamous cell carcinoma of the skin and patients 17 years old or younger were excluded. The YA included in the study were defined as individuals registered at the ICESP with cancer who were between the ages of 18 and 39 years, and older adults (OA) included in the study were defined as individuals 40 years old and older. Statistical significance was calculated using chi-square tests, and *P*<0.05 was considered significant.

## RESULTS

### Primary tumor site

**Figure 1:**
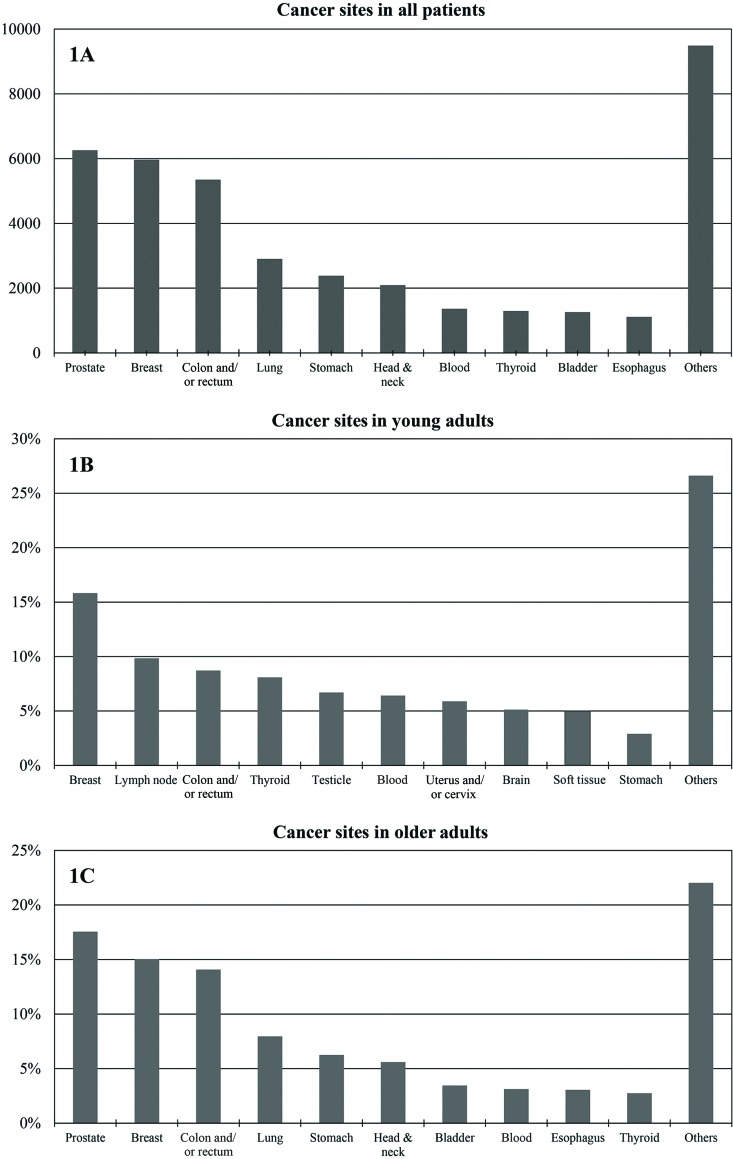
Cancer sites in all patients. **(1A)**: Most frequent tumor sites (*n*) in all patients registered at the ICESP (May 2008 to April 2014); **(1B)**: Most frequent tumors (%) in YA (18 – 39 years); 1C: Most frequent tumors (%) in older patients (≥40 years).

Between May 1^st^, 2008, and April 30^th^, 2014, 39,389 individuals with malignant neoplasms were registered at the ICESP, of whom 3,821 (9.7%) were YA (aged 18 to 39 years) and 35,568 were OA (age ≥40 years). Figure 1a shows the distribution of primary cancer sites among all patients.

Among YA, the 10 most frequent cancers were breast cancer (n=605; 15.83%), lymphoma (n=376; 9.84%), colorectal cancer (n=333; 8.71%), thyroid cancer (n=309; 8.09%), testicular cancer (n=256; 6.70%), leukemia (n=245; 6.41%), uterine cervical cancer (n=225; 5.89%), brain cancer (n=196; 5.13%), soft tissue cancers (n=154; 5.1%) and stomach cancer (n=148; 3.87%). Tumors of these types were detected in 74.5% of the patients registered. Figure 1b summarizes these findings.

For OA, the 10 most frequent cancers were prostate cancer (n=6,248; 17.57%), breast cancer (n=5,352; 15.05%), colorectal cancer (n=5,009; 14.08%), lung cancer (n=2,831; 7.96%), stomach cancer (n=2,226; 6.26%), head and neck cancer (n=1,994; 5.61%), bladder cancer (n=1,231; 3.46%), leukemia (n=1,111; 3.12%), esophageal cancer (n=1,086; 3.05%) and thyroid cancer (n=977; 2.75%). These neoplasms were present in 78.91% of the patients. Figure 1c summarizes these findings.

**Figure 2:**
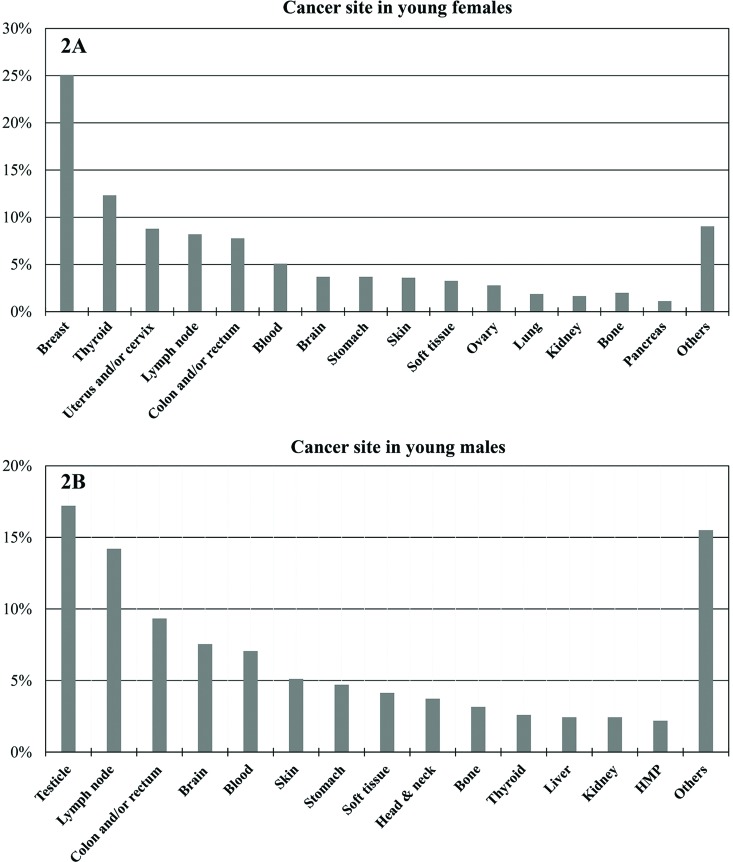
Cancer sites in young adults stratified by gender (January 2009 to December 2013). **(2A)**: primary tumor sites (%) in young adult females registered at the ICESP; **(2B)**: primary tumor sites (%) in young adult males registered at the ICESP. HMP: heart, mediastinum and pleura.

In a sample of 3,098 YA registered at the ICESP from January 1^st^, 2009, to December 31^st^, 2013, the stratification of type of cancer by gender was obtained. For YA females (n=1,866), the following primary sites were the most frequent: breast (n=468; 25%), thyroid (n=230; 12%), uterus and/or cervix (n=164; 9%), lymph node (n=153; 8%), colon and/or rectum (n=145; 8%), blood (n=95; 5%), brain (n=69; 4%), stomach (n=69; 4%), skin (n=67; 4%), soft tissue (n=61; 3%), ovary (n=2; 3%), lung (n=35; 2%), kidney (n=31; 2%), bone (n=30; 2%) and pancreas (n=25; 1%). Among the YA males (n=1,232), the most frequent primary cancer sites were as follows: testicles (n=212; 17%), lymph node (n=175; 14%), colon and/or rectum (n=115; 9%), brain (n=93; 9%), blood (n=7; 7%), skin (n=63; 5%), stomach (n=58; 5%), soft tissue (n=51; 4%), head and neck (n=46; 4%), bone (n=39; 3%), thyroid (n=32; 3%), liver (n=30; 2%), kidney (n=30; 2%) and heart, mediastinum and pleura (n=27; 2%). The graphs in Figure 2 show these results.

### Clinical stage

Approximately 13% (n=5,205) of the 39,389 patients included in this study had cancers that were not classified by the tumor-node-metastases (TNM) staging system (mostly lymphomas and leukemias), and 2.82% (n=1,114) had tumors of unknown clinical stage (CS) according to the TNM staging system. The remaining 35,335 individuals had neoplasms classified as CS 0 (n=710; 2%), CS I (n=5,375; 14%), CS II (n=9,535; 24%), CS III (n=8,047; 20%) or CS IV (n=11,668; 30%).

**Figure 3:**
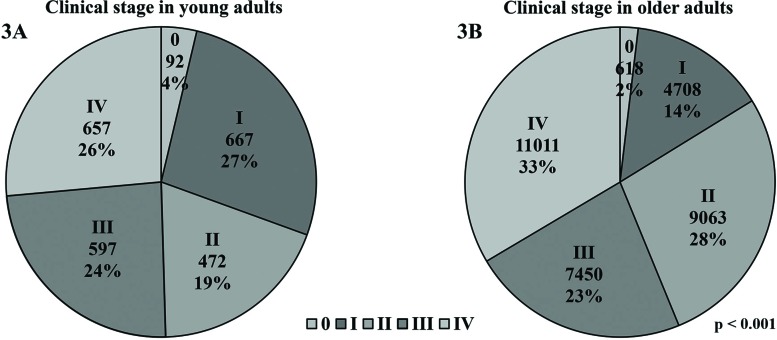
Clinical stage according to the TNM staging system. **(3A)**: CS of all young adults (18-39 years) registered at the ICESP; **(3B)**: CS of all older adults registered at the ICESP (≥40 years).

Approximately half of the YA were diagnosed with advanced and metastatic disease (24% with CS III and 26.44% with CS IV). In addition, 19% of the YA had tumors classified as CS II, 26.84% had tumors classified as CS I, and 3.7% of the YA had *in situ* disease (CS 0). A significantly different distribution was observed in the OA, among whom only 1.88% were diagnosed with *in situ* neoplasms, 14.33% had tumors classified as CS I, 27.6% had tumors classified as CS II, 22.68% had tumors classified as CS III and 32.52% had metastatic disease (*p*<0.001). These results are represented in Figure 3.

**Figure 4:**
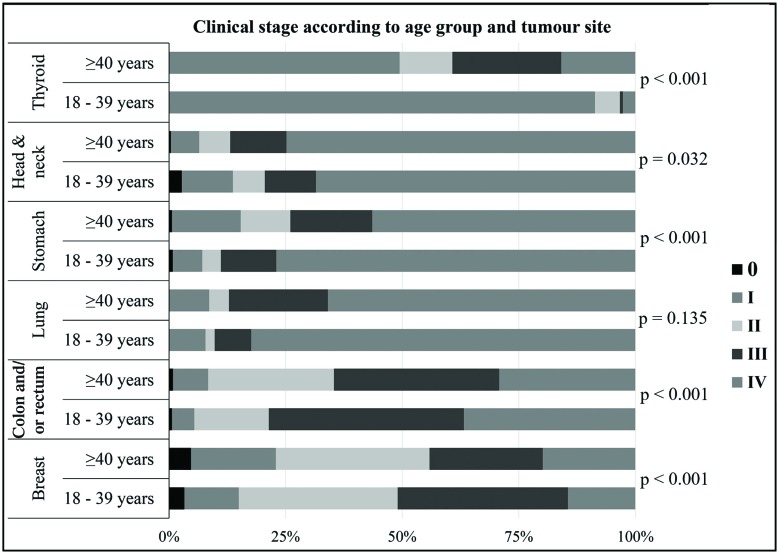
Clinical stage according to age and tumor site.

Clinical stage classification was evaluated for the following six tumor sites that were frequently found in both the YA and the OA: breast, colon and/or rectum, lung, stomach, head and neck and thyroid. Compared with the OA, the YA presented with advanced and metastatic disease (CS III and IV) more frequently when they had breast (YA 51% *vs*. OA 44.1%; *p*=0.002), colorectal (YA 78.6% *vs*. 64.6%; *p*<0.001) and stomach tumors (YA 88.9% *vs* OA 74.0%; *p*<0.001). In contrast, compared with the YA, the OA were more frequently diagnosed as having cancer classified as CS III and IV when they had thyroid cancer (YA 3.3% *vs.* OA 39.2%; *p*<0.001). Among the patients with lung cancer, the YA had significantly more metastatic disease than the OA (YA 82.4% *vs* OA 65.9%; *p*=0.014). No significant difference in CS at diagnosis was found in patients with head and neck cancers. Figure 4 and Table 1 show these results.

### Histological type

The histological classifications of breast, colorectal, uterine cervical, stomach and lung tumors were retrieved for the YA. Among the YA with breast cancer, the most common histological type was infiltrating ductal carcinoma (86.12%), followed by *in situ* ductal carcinoma (2.81%) and lobular carcinoma (1.82%). The most commonly diagnosed colorectal tumors were adenocarcinomas not otherwise specified (NOS) (45.35%), tubular adenocarcinoma (30.93%) and mucinous adenocarcinoma (9.61%). The most common uterine cervical cancer histological subtypes were squamous cell carcinoma NOS (65.26%), squamous intraepithelial neoplasms grade III (20.53%) and adenocarcinoma NOS (9.47%). In YA with stomach neoplasms, most tumors were classified as signet ring cell adenocarcinomas (49.32%), adenocarcinomas NOS (19.59%) and tubular adenocarcinomas (6.08%). Finally, the most commonly diagnosed lung cancer histological subtypes in the YA were adenocarcinoma NOS (50.79%), squamous cell carcinoma NOS (6.35%), undifferentiated carcinoma NOS (6.35%) and carcinoid tumor NOS (6.35%). Table 2 summarizes these findings.

## DISCUSSION

Cancer in young adults represents a special challenge for patients, family members and physicians. YA have specific demands with regard to their medical, psychological and social care to ensure appropriate treatment and reintegration into their social lives and the work force 4,5. Information regarding the most common types of neoplasms in YA is scarce in Brazil, and this study aimed to evaluate the frequency of various types of cancers diagnosed in a population from a large reference cancer hospital in Brazil.

It is noteworthy that the incidence rates of the most frequently treated cancers at the ICESP closely reflect the incidence rates of those cancers in Brazil. According to the INCA projection for 2018 9, the most common cancers in Brazil are prostate, breast, colorectal, lung, stomach, uterine cervical, oral cavity, central nervous system, and esophageal cancers as well as leukemias. The main differences between the most common cancers in Brazil and those at the ICESP are that uterine cervical and central nervous system neoplasms are not among the 10 most frequently treated cancers at the ICESP. In the case of uterine cervical neoplasms, this difference might be explained by this type of cancer being less frequently diagnosed in more developed regions, such as São Paulo, than in the less developed regions of the country 10. In addition, at the ICESP, thyroid and bladder cancer are among the 10 most frequently treated cancers, even though they are not included among the top ten most common cancers in Brazil. It is important to point out that our study aimed to evaluate the most frequently treated cancers in a cancer hospital and not the national incidence rate; therefore, this observation may reflect a selection bias due to peculiarities of the study.

In 2016, the INCA published a comprehensive study focusing on the incidence, mortality and hospital morbidity rates in AYA aged 15 to 29 years 8. Incidence data were obtained from 25 out of the existing 31 RCBPs. In Brazil, the median of the mean incidence rates for AYA (15 to 29 years old) adjusted for age was 236.16 per million (241.74 per million in females and 212.71 per million in males) in the reference period, according to data from 24 RCBPs. Among all tumors diagnosed, 4.3% occurred in AYA, which is lower than our finding of 9.7%. However, a higher age limit was adopted in our study (18 to 39 years) than in the INCA data (15 to 29 years), as we assumed the previously adopted recommendations regarding the maximum age cutoff value for the definition of YA 4.

The most frequent neoplasms in AYA ages 15-29 years reported by the INCA were carcinomas of all types (34.2%); followed by lymphomas (12.1%); skin tumors, including melanomas and carcinomas (9.0%); leukemias (8.0%), neoplasms NOS (7.3%); central nervous system cancers (6.9%); germ cell neoplasms (5.3%); soft tissue sarcomas (5.1%); bone tumors (3.8%), and miscellaneous cancers (0.9%). These results are in agreement with those attained using the data from the ICESP; if we had grouped breast, colorectal, thyroid and stomach cancer together, they would account for a total of 36.5% of all cancers.

At the ICESP, YA were generally diagnosed with less advanced disease than older adults. This result is possibly influenced by thyroid cancer; more than 85% of YA with thyroid cancer are diagnosed with CS 0 and I. Furthermore, this general finding is reversed when investigating other carcinomas frequently found in all age groups. For breast, colorectal, stomach and lung cancers, YA are more frequently diagnosed with advanced and/or metastatic disease than OA. Previous studies reported similar findings 6,11-14, and this trend towards diagnosis at later stages may affect the higher morbidity and mortality rates observed in YA with carcinomas compared with OA with carcinomas. In addition, diagnosis at an advanced stage of the disease raises the suspicion of a delayed diagnosis due to the underestimation of the signs and symptoms, possibly resulting from the relatively low incidence of cancer in YA and a low awareness among health professionals of the incidence of cancer in this age group 5,6. For breast cancer, specifically, age is considered a poor prognostic factor independent of stage 13, whereas for lung cancer, YA tend to have better prognoses in all stages compared with OA 13,14.

Regarding the histological information, patients registered at the ICESP had similar patterns as those previously described. Although squamous cell carcinoma was the most frequent histological type, due to its close association with HPV infection in the uterus and cervix, the incidence of adenocarcinoma has been increasing worldwide and accounts for 10 to 15% of cases 15. Similar proportions were found in our population. For stomach cancer, diffuse and intestinal subtypes are more frequently detected in YA and OA, respectively 11,16. Our data substantiate this, as signet ring cell carcinoma, a type highly associated with diffuse histology, represented almost half of the gastric tumors diagnosed in YA in this study. The most common histological type of lung cancer in our study was adenocarcinoma (50.79%), followed by squamous cell carcinoma (6.35%), which was also similar to the results of previous reports 14,17,18. In addition, most breast tumors were infiltrating ductal carcinomas, and only a small proportion were lobular carcinomas (1.82%). This observation is in agreement with the findings of other studies that demonstrated that the frequency of lobular carcinoma is lower in young women than in older women 19,20.

Although we did not estimate incidence rates, it is interesting to observe that, according to the INCA 6, the trend in the AAPC in the incidence rates of carcinomas in the reference period in YA aged 15-29 years tended to increase in eight out of the 12 PBCRs evaluated and to decrease in only one PBCR. In Brazil, the mean mortality rate adjusted for age for all neoplasms in AYA was 66.97 per million in both sexes (72.08 per million in males and 61.72 per million in females). Notably, even though the AAPC in mortality rates did not vary for all cancers, a small decrease in the period from 1982 to 1983 was observed, followed by increases in the periods from 1992 to 1998 and from 1998 to 2013.

To the best of our knowledge, this study is the first to evaluate the clinical stages and histological types of common carcinomas in YA in Brazil. The limitations of this study include a small sample size of YA per type of cancer and the use of a single hospital database. However, our study revealed tumor-specific information regarding cancer in YA, which may be valuable in the clinical setting. As YA tend to present advanced stage carcinomas, future studies are needed to investigate the reasons for the delay in diagnosis. In summary, improved understanding of the unique characteristics of cancer in this age group is important to reduce its high biological, social and psychological impacts.

Data regarding cancer in YA is still scarce. Our results revealed that YA may be diagnosed with cancers at more advanced stages, indicating that health professionals should be aware of the incidence rates of neoplasms in this age group. It is important to better understand cancer in YA and to implement dedicated healthcare strategies addressing the needs of these patients.

## AUTHOR CONTRIBUTIONS

Cormedi MC interpreted the data, wrote the manuscript and generated the figures. Lopes EF collected and interpreted the data and generated the tables. Roela RA and Maistro S interpreted the data and configured the tables. Folgueira MA designed and supervised the research, interpreted and discussed the data, and wrote and submitted the manuscript. All authors reviewed and approved the manuscript.

## Figures and Tables

**Table 1:** Clinical stage according to tumor site and age group.

Primary tumor site	CS	Young adults		Older adults		*p*
		N	%	N	%	
Breast	0	19	3.30	247	4.71	<0.001
	I	67	11.65	954	18.21	
	II	196	34.09	1726	32.95	
	III	210	36.52	1272	24.28	
	IV	83	14.43	1040	19.85	
	Total	575	100.00	5239	100.00	
Colon and/or rectum	0	2	0.64	42	0.87	<0.001
	I	15	4.79	364	7.53	
	II	50	15.97	1302	26.95	
	III	131	41.85	1714	35.48	
	IV	115	36.74	1409	29.17	
	Total	313	100.00	4831	100.00	
Lung	0	0	0.00	3	0.11	0.135
	I	4	7.84	233	8.51	
	II	1	1.96	116	4.24	
	III	4	7.84	581	21.23	
	IV	42	82.35	1804	65.91	
	Total	51	100.00	2737	100.00	
Stomach	0	1	0.79	13	0.64	<0.001
	I	8	6.35	299	14.74	
	II	5	3.97	216	10.65	
	III	15	11.90	356	17.55	
	IV	97	76.98	1144	56.41	
	Total	126	100.00	2028	100.00	
Head and neck	0	2	2.74	8	0.42	0.032
	I	8	10.96	115	6.06	
	II	5	6.85	126	6.64	
	III	8	10.96	229	12.07	
	IV	50	68.49	1419	74.80	
	Total	73	100.00	1897	100.00	
Thyroid	I	275	91.36	464	49.47	<0.001
	II	16	5.32	106	11.30	
	III	2	0.66	219	23.35	
	IV	8	2.66	149	15.88	
	Total	301	100.00	938	100.00	

**Table 2:** Tumor histological types in young adults.

Primary tumor site	Histological type	N	%
Breast	Infiltrating ductal carcinoma	521	86.12
	*In situ* intraductal carcinoma	17	2.81
	Lobular carcinoma	11	1.82
	Phyllodes malignant tumor	8	1.32
	Acinic cell carcinoma	6	0.99
	Others	42	6.94
	Total	605	100.00
Colon and/or rectum	Adenocarcinoma NOS	151	45.35
	Tubular adenocarcinoma	103	30.93
	Mucinous adenocarcinoma	32	9.61
	Signet ring cell adenocarcinoma	11	3.30
	Squamous cell carcinoma NOS	11	3.30
	Neuroendocrine carcinoma NOS	10	3.00
	Others	15	4.50
	Total	333	100.00
Uterus and/or cervix	Squamous cell carcinoma NOS	124	65.26
	Squamous intraepithelial neoplasms grade III	39	20.53
	Adenocarcinoma NOS	18	9.47
	Squamous cell *in situ* NOS	11	5.79
	Mucinous adenocarcinoma	5	2.63
	Adenocarcinoma *in situ* NOS	4	2.11
	Others	24	12.63
	Total	190	100.00
Stomach	Signet ring cell adenocarcinoma	73	49.32
	Adenocarcinoma NOS	29	19.59
	Tubular adenocarcinoma	9	6.08
	Mucinous adenocarcinoma	7	4.73
	Gastrointestinal stromal sarcomas	7	4.73
	Malignant lymphoma diffuse large cell NOS	5	3.38
	Others	18	12.16
	Total	148	100.00
Lung	Adenocarcinoma NOS	32	50.79
	Squamous cell carcinoma NOS	4	6.35
	Undifferentiated carcinoma NOS	4	6.35
	Carcinoid tumors NOS	4	6.35
	Bronchioloalveolar carcinoma NOS	3	4.76
	Neuroendocrine carcinoma NOS	3	4.76
	Small cell carcinoma NOS	2	3.17
	Others	11	17.46
	Total	63	100.00

NOS: not otherwise specified.
